# Optimal Fusion Estimation with Multi-Step Random Delays and Losses in Transmission

**DOI:** 10.3390/s17051151

**Published:** 2017-05-18

**Authors:** Raquel Caballero-Águila, Aurora Hermoso-Carazo, Josefa Linares-Pérez

**Affiliations:** 1 Dpto. de Estadística, Universidad de Jaén, Paraje Las Lagunillas, 23071 Jaén, Spain; 2 Dpto. de Estadística, Universidad de Granada, Avda. Fuentenueva, 18071 Granada, Spain; ahermoso@ugr.es (A.H.-C.); jlinares@ugr.es (J.L.-P.)

**Keywords:** recursive fusion estimation, sensor networks, random parameter matrices, random delays, packet dropouts

## Abstract

This paper is concerned with the optimal fusion estimation problem in networked stochastic systems with bounded random delays and packet dropouts, which unavoidably occur during the data transmission in the network. The measured outputs from each sensor are perturbed by random parameter matrices and white additive noises, which are cross-correlated between the different sensors. Least-squares fusion linear estimators including filter, predictor and fixed-point smoother, as well as the corresponding estimation error covariance matrices are designed via the innovation analysis approach. The proposed recursive algorithms depend on the delay probabilities at each sampling time, but do not to need to know if a particular measurement is delayed or not. Moreover, the knowledge of the signal evolution model is not required, as the algorithms need only the first and second order moments of the processes involved. Some of the practical situations covered by the proposed system model with random parameter matrices are analyzed and the influence of the delays in the estimation accuracy are examined in a numerical example.

## 1. Introduction

Over the last few decades, research on the estimation problem for networked stochastic systems has gained considerable attention, due to the undeniable advantages of networked systems, whose applicability is encouraged, among other causes, by the development and advances in communication technology and the growing use of wireless networks. As it is well known, the Kalman filter provides a recursive algorithm for the optimal least-squares estimator in stochastic linear systems, assuming that the system model is exactly known and all of the measurements are instantly updated. The development of sensor networks motivates the necessity of designing new estimation algorithms that integrate the information of all the sensors to achieve a satisfactory performance; thus, using different fusion techniques, the measurements from multiple sensors are combined to obtain more accurate estimators than those obtained when a single sensor is used. In this framework, important extensions of the Kalman filter have been proposed for conventional sensor networks in which the measured outputs of the sensors always contain the actual signal contaminated by additive noises, and the transmissions are carried through perfect connections (see, e.g., [1,2,3] and references therein).

However, in a network environment, usually the standard observation models are not suitable due to the existence of network-induced uncertainties that can occur in both of the sensor measured outputs, and during the data transmission through the network. Accordingly, the consideration of appropriate observation models is vitally important to address the estimation problem in networked systems. Random failures in the transmission of measured data, together with the inaccuracy of the measurement devices, cause often the degradation of the estimator performance in networked systems. In light of these concerns, the estimation problem with one or even several network-induced uncertainties is recently attracting considerable attention, and the design of new fusion estimation algorithms has become an active research topic (see, e.g., [4,5,6,7,8,9,10,11,12,13] and references therein). In addition, some recent advances on the estimation, filtering and fusion for networked systems with network-induced phenomena can be reviewed in [14,15], where a detailed overview of this field is presented.

One of the most common network-induced uncertainties in the measured outputs of the different sensors is the presence of multiplicative noise, due to different reasons, such as interferences or intermittent sensor failures. Specifically, in situations involving random observation losses (see [5]), sensor gain degradation (see [6]), missing or fading measurements (see [13,16], respectively), the sensor observation equations include multiplicative noises. A unified framework to model these random phenomena is provided by the use of random measurement matrices in the sensor observation model. For this reason, the estimation problem in networked systems with random measurement matrices has become a fertile research subject, since this class of systems allow for covering different networked-induced random uncertainties as those mentioned above (see, e.g., [17,18,19,20,21,22,23], and references therein).

In relation to the network-induced uncertainties during the data transmission, it must be indicated that sudden changes in the environment and the unreliability of the communication network, together with the limited bandwidths of communication channels, cause unavoidable random failures during the transmission process. Generally, random communication delays and/or transmission packet dropouts are two essential issues that must be taken into account to model the measurements, which, being available after transmission, will be used for the estimation. Several estimation algorithms have been proposed in multisensor systems considering either transmission delays or packet losses (see, e.g., [24,25]) and also taking into account random delays and packet dropouts simultaneously (see, e.g., [4,23,26]). By using the state augmentation method, systems with random delays and packet dropouts can be transformed into systems with random parameter matrices (see, e.g., [8,9,10,20]). Hence, systems with random parameter measurement matrices also provide an appropriate unified context for modelling these random phenomena in the transmission.

Nevertheless, it must be indicated that the state augmentation method leads to a rise of the computational burden, due to the increase of the state dimension. Actually, in models with more than one or two-step random delays, the computational cost can be excessive and alternative ways to model and address the estimation problem in this class of systems need to be investigated. Recently, a great variety of models have been used to describe the phenomena of multi-step random delays and packet losses during the data transmission in networked systems, and fusion estimation algorithms have been proposed based on different approaches—for example, the recursive matrix equation method in [6], the measurement reorganisation approach in [27], the innovation analysis approach in [28] and the state augmentation approach in [29,30,31]. It should be noted that, in the presence of multi-step random delays and packet losses during the data transmission, many difficulties can arise in the design of the optimal estimators when the state augmentation approach is not used.

In view of the above considerations, this paper is concerned with the optimal fusion estimation problem, in the least-squares linear sense, for sensor networks featuring stochastic uncertainties in the sensor measurements, together with multi-step random delays and packet dropouts during the data transmission. The derivation of the estimation algorithms will be carried out without using the evolution model generating the signal process. The uncertainties in the measured outputs of the different sensors are described by random measurement matrices. The multi-step random delays in the transmissions are modeled by using a collection of Bernoulli sequences with known distributions and different characteristics at each sensor; the exact value of these Bernoulli variables is not required, and only the information about the probability distribution is needed. To the best of the authors’ knowledge, the optimal estimation problem (including prediction, filtering and fixed-point smoothing) has not been investigated for systems involving random measurement matrices and transmission multi-step random delays simultaneously, and, therefore, it constitutes an interesting research challenge. The main contributions of this research can be highlighted as follows: (a) even though our approach, based on covariance information, does not require the signal evolution model, the proposed algorithms are also applicable in situations based on the state-space model (see Remark 1); (b) random measurement matrices are considered in the measured outputs, thus providing a unified framework to address different network-induced phenomena (see Remark 2); (c) besides the stochastic uncertainties in the sensor measurements, simultaneous multi-step random delays and losses with different rates are considered in the data transmission; (d) unlike most papers about multi-step random delays, in which only the filtering problem is considered, we propose recursive algorithms for the prediction, filtering an fixed-point smoothing estimators under the innovation approach, which are computationally very simple and suitable for online applications; and (e) optimal estimators are obtained without using the state augmentation approach, thus reducing the computational cost in comparison with the augmentation method.

The rest of the paper is organized as follows. In Section 2, we present the sensor network and the assumptions under which the optimal linear estimation problem will be addressed. In Section 3, the observation model is rewritten in a compact form and the innovation approach to the least-squares linear estimation problem is formulated. In Section 4, recursive algorithms for the prediction, filtering and fixed-point smoothing estimators are derived. A simulation example is given in Section 5 to show the performance of the proposed estimators. Finally, some conclusions are drawn in Section 6.

**Notations.**  The notations used throughout the paper are standard. $R^{n}$ and $R^{m\times n}$ denote the *n*-dimensional Euclidean space and the set of all m×n real matrices, respectively. For a matrix *A*, $A^{T}$ and $A^{-1}$ denote its transpose and inverse, respectively. The shorthand $Diag(A_{1},\ldots ,A_{m})$ stands for a block-diagonal matrix whose diagonal matrices are $A_{1},\ldots ,A_{m}$. $1_{n}={\left(1,\ldots ,1\right)}^{T}$ denotes the all-ones n×1-vector and $I_{n}$ represent the n×n-identity matrix. If the dimensions of matrices are not explicitly stated, they are assumed to be compatible with algebraic operations. The Kronecker and Hadamard product of matrices will be denoted by ⊗ and ∘, respectively. $\delta_{k,s}$ denotes the Kronecker delta function. For any a,b∈R, a∧b is used to mean the minimum of *a* and *b*.

## 2. Observation Model and Preliminaries

In this paper, the optimal fusion estimation problem of multidimensional discrete-time random signals from measurements obtained by a sensor network is addressed. At each sensor, the measured outputs are perturbed by random parameter matrices and white additive noises that are cross-correlated at the same sampling time between the different sensors. The estimation is performed in a processing centre connected to all sensors, where the complete set of sensor data is combined, but due to eventual communication failures, congestion or other causes, random delays and packet dropouts are unavoidable during the transmission process. To reduce the effect of such delays and packet dropouts without overloading the network traffic, each sensor measurement transmits a fixed number of consecutive sampling times and, when several packets arrive at the same time, the receiver discards the oldest ones, so that only one measured output is processed for the estimation at each sampling time.

### 2.1. Signal Process

The optimal estimators will be obtained using the least-squares (LS) criterion and without requiring the evolution model generating the signal process. Actually, the proposed estimation algorithms, based on covariance information, only need the mean vectors and covariance functions of the processes involved, and the only requirement will be that the signal covariance function must be factorizable according to the following assumption:

(A1) The $n_{x}$-dimensional signal process $\left\{x_{k};\;k\geq 1\right\}$ has zero mean and its autocovariance function is expressed in a separable form, $E\left[x_{k}x_{s}^{T}\right]=A_{k}B_{s}^{T},\;s\leq k,$ where $A_{k},B_{s}\in R^{n_{x}\times n}$ are known matrices.

**Remark 1** (on assumption (A1)). *The estimation problems based on the state-space model require new estimation algorithms when the signal evolution model is modified; therefore, the algorithms designed for stationary signals driven by $x_{k+1}=\Phi x_{k}+\xi_{k}$ cannot be applied for non-stationary signals generated by $x_{k+1}=\Phi_{k}x_{k}+\xi_{k}$, and these, in turn, cannot be used in uncertain systems where $x_{k+1}=\left(\Phi_{k}+\epsilon_{k}{\hat{\Phi }}_{k}\right)x_{k}+\xi_{k}$. A great advantage of assumption* (A1) *is that it covers situations in which the signal evolution model is known, for both stationary and non-stationary signals (see, e.g., [23]). In addition, in uncertain systems with state-dependent multiplicative noise, as those considered in [6,32], the signal covariance function is factorizable, as it is shown in Section 5. Hence, assumption* (A1) *on the signal autocovariance function provides a unified context to deal with different situations based on the state-space model, avoiding the derivation of specific algorithms for each of them.*

### 2.2. Multisensor Observation Model

Assuming that there are *m* different sensors, the measured outputs before transmission, $z_{k}^{\left(i\right)}\in R^{n_{z}}$, are described by the following observation model:

(1) $$z_{k}^{\left(i\right)}=H_{k}^{\left(i\right)}x_{k}+v_{k}^{\left(i\right)},\;\;k\geq 1;\;\;\;i=1,\ldots ,m,$$

where the measurement matrices, $H_{k}^{\left(i\right)}$, and the noise vectors, $v_{k}^{\left(i\right)}$, satisfy the following assumptions:

(A2) $\left\{H_{k}^{\left(i\right)};\;k\geq 1\right\}$, i=1,…,m, are independent sequences of independent random parameter matrices, whose entries have known means and second-order moments; we will denote ${\bar{H}}_{k}^{\left(i\right)}\equiv E\left[H_{k}^{\left(i\right)}\right],\;k\geq 1$.
(A3) $\left\{v_{k}^{\left(i\right)};\;k\geq 1\right\},\;i=1,\ldots ,m$, are white noise sequences with zero mean and known second-order moments, satisfying $E\left[v_{k}^{\left(i\right)}v_{s}^{(j)T}\right]=R_{k}^{\left(ij\right)}\delta_{k,s},\;\;i,j=1,\ldots ,m.$

**Remark 2** (on assumption (A2)). *Usually, in network environments, the measurements are subject to different network-induced random phenomena and new estimation algorithms must be designed to incorporate the effects of these random uncertainties. For example, in systems with stochastic sensor gain degradation or missing measurements as those considered in [6,7], respectively, or in networked systems involving stochastic multiplicative noises in the state and measurement equations (see, e.g., [31,32]), new estimation algorithms are proposed since the conditions necessary to implement the conventional ones are not met. The aforementioned systems are particular cases of systems with random measurement matrices, and, hence, assumption* (A2) *allows for designing a unique estimation algorithm, which is suitable to address all of these situations involving random uncertainties. In addition, based on an augmentation approach, random measurement matrices can be used to model the measured outputs of sensor networks with random delays and packet dropouts (see, e.g., [8,9,10,20]). Therefore, assumption* (A2) *provides a unified framework to deal with a great variety of network-induced random phenomena as those mentioned above.*

### 2.3. Measurement Model with Transmission Random Delays and Packet Losses

Assuming that the maximum time delay is *D*, the measured output of the *i*-th sensor at time *r*, $z_{r}^{\left(i\right)}$, is transmitted during the sampling times r,r+1,⋯,r+D, but, at each sampling time k>D, only one of the measurements $z_{k-D}^{\left(i\right)},\ldots ,z_{k}^{\left(i\right)}$ is processed. Consequently, at any time k>D, the measurement processed can either arrive on time or be delayed by d=1,…,D sampling periods, while at any time k≤D, the measurement processed can be delayed only by d=1,…,k−1 sampling periods, since only $z_{1}^{\left(i\right)},\ldots ,z_{k}^{\left(i\right)}$ are available. Assuming, moreover, that the transmissions are perturbed by additive noises, the measurements received at the processing centre, impaired by random delays and packet losses, can be described by the following model:

(2) $$y_{k}^{\left(i\right)}=\sum_{d=0}^{(k-1)\wedge D}\gamma_{d,k}^{\left(i\right)}z_{k-d}^{\left(i\right)}+w_{k}^{\left(i\right)},\;\;k\geq 1,$$

where the following assumptions on the random variables modelling the delays, $\gamma_{d,k}^{\left(i\right)}$, and the transmision noise, $w_{k}^{\left(i\right)}$, are required:

(A4) For each d=0,1,…,D, $\left\{\gamma_{d,k}^{\left(i\right)};\;k>d\right\}$, i=1,…,m, are independent sequences of independent Bernoulli random variables with $P\left[\gamma_{d,k}^{\left(i\right)}=1\right]={\bar{\gamma }}_{d,k}^{\left(i\right)}$ and $\sum_{d=0}^{(k-1)\wedge D}\;\;\gamma_{d,k}^{\left(i\right)}\leq 1,\;k\geq 1.$
(A5) $\left\{w_{k}^{\left(i\right)};\;k\geq 1\right\},\;i=1,\ldots ,m$, are white noise sequences with zero mean and known second-order moments, satisfying $E\left[w_{k}^{\left(i\right)}w_{s}^{(j)T}\right]=Q_{k}^{\left(ij\right)}\delta_{k,s},\;\;i,j=1,\ldots ,m.$

**Remark 3** (on assumption (A4)). For i=1,…,m, when $\gamma_{0,k}^{\left(i\right)}=1$, the transmission of the i-th sensor is perfect and neither delay nor loss occurs at time k; that is, with probability ${\bar{\gamma }}_{0,k}^{\left(i\right)}$, the k-th measurement of the i-th sensor is received and processed on time. Since $\sum_{d=0}^{(k-1)\wedge D}\;\;\gamma_{d,k}^{\left(i\right)}\leq 1$, if $\gamma_{0,k}^{\left(i\right)}=0$, there then exists at most one d=1,…,D, such that $\gamma_{d,k}^{\left(i\right)}=1$. If there exists d such that $\gamma_{d,k}^{\left(i\right)}=1$ (which occurs with probability ${\bar{\gamma }}_{d,k}^{\left(i\right)}$), then the measurement is delayed by d sampling periods. Otherwise, $\gamma_{d,k}^{\left(i\right)}=0$ for all d, meaning that the measurement gets lost during the transmission at time k with probability $1-\;\sum_{d=0}^{(k-1)\wedge D}\;\;{\bar{\gamma }}_{d,k}^{\left(i\right)}$.

Finally, the following independence hypothesis is assumed:

(A6) For i=1,…,m and d=0,1,…,D, the processes $\left\{x_{k};\;k\geq 1\right\}$, $\left\{H_{k}^{\left(i\right)};\;k\geq 1\right\}$, $\left\{v_{k}^{\left(i\right)};\;k\geq 1\right\}$, $\left\{w_{k}^{\left(i\right)};\;k\geq 1\right\}$ and $\left\{\gamma_{d,k}^{\left(i\right)};\;k>d\right\}$ are mutually independent.

## 3. Problem Statement

Given the observation Equations (1) and (2) with random measurement matrices and transmission random delays and packet dropouts, our purpose is to find the LS linear estimator, ${\hat{x}}_{k/L}$, of the signal $x_{k}$ based on the observations from the different sensors $\left\{y_{1}^{\left(i\right)},\ldots ,y_{L}^{\left(i\right)},\;i=1,\ldots ,m\right\}$. Specifically, our aim is to obtain recursive algorithms for the predictor (L<k), filter (L=k) and fixed-point smoother (*k* fixed and L>k).

### 3.1. Stacked Observation Model

Since the measurements coming from the different sensors are all gathered and jointly processed at each sampling time *k*, we will consider the vector constituted by the meaurements from all sensors, $y_{k}={\left(y_{k}^{(1)T},\ldots ,y_{k}^{(m)T}\right)}^{T}$. More specifically, the observation Equations (1) and (2) of all sensors are combined, yielding the following stacked observation model:

(3) $$\begin{array}{c} z_{k}=H_{k}x_{k}+v_{k},\;\;k\geq 1,\\ y_{k}=\sum_{d=0}^{(k-1)\wedge D}\Gamma_{d,k}z_{k-d}+w_{k},\;\;k\geq 1, \end{array}$$

where $z_{k}={\left(z_{k}^{(1)T},\ldots ,z_{k}^{(m)T}\right)}^{T}$, $H_{k}={\left(H_{k}^{(1)T},\ldots ,H_{k}^{(m)T}\right)}^{T}$, $v_{k}={\left(v_{k}^{(1)T},\ldots ,v_{k}^{(m)T}\right)}^{T}$, $w_{k}={\left(w_{k}^{(1)T},\ldots ,w_{k}^{(m)T}\right)}^{T}$ and $\Gamma_{d,k}=Diag\left(\gamma_{d,k}^{\left(1\right)},\ldots ,\gamma_{d,k}^{\left(m\right)}\right)⊗I_{n_{z}}$.

Hence, the problem is to obtain the LS linear estimator of the signal, $x_{k}$, based on the measurements $\left\{y_{1},\ldots ,y_{L}\right\}$, given in the observation Equation (3). Next, we present the statistical properties of the processes involved in Equation (3), from which the LS estimation algorithms of the signal will be derived; these properties are easily inferred from the assumptions (A1)–(A6).

(P1) $\left\{H_{k};\;k\geq 1\right\}$ is a sequence of independent random parameter matrices with known means, ${\bar{H}}_{k}\equiv E\left[H_{k}\right]={\left({\bar{H}}_{k}^{(1)T},\ldots ,{\bar{H}}_{k}^{(m)T}\right)}^{T}$, and
  $$\begin{array}{cc} E\left[H_{k}x_{k}x_{s}^{T}H_{s}^{T}\right]=E\left[H_{k}A_{k}B_{s}^{T}H_{s}^{T}\right]={\left(E[H_{k}^{\left(i\right)}A_{k}B_{s}^{T}H_{s}^{(j)T}]\right)}_{i,j=1\ldots ,m} & ,\;\;s\leq k, \end{array}$$
  where $E\left[H_{k}^{\left(i\right)}A_{k}B_{s}^{T}H_{s}^{(j)T}\right]={\bar{H}}_{k}^{\left(i\right)}A_{k}B_{s}^{T}{\bar{H}}_{s}^{(j)T}$, for j≠i or s≠k, and the entries of $E\left[H_{k}^{\left(i\right)}A_{k}B_{k}^{T}H_{k}^{(i)T}\right]$ are computed as follows:
  $${\left(E\left[H_{k}^{\left(i\right)}A_{k}B_{k}^{T}H_{k}^{(i)T}\right]\right)}_{\;pq}=\sum_{a=1}^{n_{x}}\sum_{b=1}^{n_{x}}E\left[h_{pa}^{\left(i\right)}\left(k\right)h_{qb}^{\left(i\right)}\left(k\right)\right]{\left(A_{k}B_{k}^{T}\right)}_{ab},\;\;p,q=1,\ldots ,n_{z},$$
  where $h_{pq}^{\left(i\right)}\left(k\right)$ denotes the (p,q)−entry of the matrix $H_{k}^{\left(i\right)}$.
(P2) The noises $\left\{v_{k};\;k\geq 1\right\}$ and $\left\{w_{k};\;k\geq 1\right\}$ are zero-mean sequences with known second-order moments given by the matrices $R_{k}\equiv {\left(R_{k}^{\left(ij\right)}\right)}_{i,j=1,\ldots ,m}$ and $Q_{k}\equiv {\left(Q_{k}^{\left(ij\right)}\right)}_{i,j=1,\ldots ,m}$.
(P3) $\left\{\Gamma_{d,k};\;k>d\right\}$, d=0,1,…,D, are sequences of independent random matrices with known means, ${\bar{\Gamma }}_{d,k}\equiv E\left[\Gamma_{d,k}\right]=Diag\left({\bar{\gamma }}_{d,k}^{\left(1\right)},\ldots ,{\bar{\gamma }}_{d,k}^{\left(m\right)}\right)⊗I_{n_{z}}$, and if we denote $\gamma_{d,k}={\left(\gamma_{d,k}^{\left(1\right)},\ldots ,\gamma_{d,k}^{\left(m\right)}\right)}^{T}⊗1_{n_{z}}$ and ${\bar{\gamma }}_{d,k}=E\left[\gamma_{d,k}\right]$, the covariance matrices $\Sigma_{d,d^{\prime }}^{\gamma_{k}}\equiv E\left[\left(\gamma_{d,k}-{\bar{\gamma }}_{d,k}\right){\left(\gamma_{d^{\prime },k}-{\bar{\gamma }}_{d^{\prime },k}\right)}^{T}\right]$, for $d,d^{\prime }=0,1,\ldots ,D$, are also known matrices. Specifically,
  (4) $$\Sigma_{d,d^{\prime }}^{\gamma_{k}}=Diag\left(Cov\left(\gamma_{d,k}^{\left(1\right)},\gamma_{d^{\prime },k}^{\left(1\right)}\right),\ldots ,Cov\left(\gamma_{d,k}^{\left(m\right)},\gamma_{d^{\prime },k}^{\left(m\right)}\right)\right)⊗\left(1_{n_{z}}1_{n_{z}}^{T}\right),$$
  with $Cov\left(\gamma_{d,k}^{\left(i\right)},\gamma_{d^{\prime },k}^{\left(i\right)}\right)=\left\{\begin{array}{cc} {\bar{\gamma }}_{d,k}^{\left(i\right)}\left(1-{\bar{\gamma }}_{d,k}^{\left(i\right)}\right), & d^{\prime }=d,\\ -{\bar{\gamma }}_{d,k}^{\left(i\right)}{\bar{\gamma }}_{d^{\prime },k}^{\left(i\right)}, & \;d^{\prime }\neq d. \end{array}\right.$ Moreover, for any deterministic matrix *S*, the Hadamard product properties guarantee that
  $$E\left[\left(\Gamma_{d,k}-{\bar{\Gamma }}_{d,k}\right)S\left(\Gamma_{d^{\prime }\;,k}-{\bar{\Gamma }}_{d^{\prime }\;,k}\right)\right]=\Sigma_{d,d^{\prime }}^{\gamma_{k}}\circ S.$$
(P4) For d=0,1,…,D, the signal, $\left\{x_{k};\;k\geq 1\right\}$, and the processes $\left\{H_{k};\;k\geq 1\right\}$, $\left\{v_{k};\;k\geq 1\right\}$, $\left\{w_{k};\;k\geq 1\right\}$ and $\left\{\Gamma_{d,k};\;k>d\right\}$ are mutually independent.

**Remark 4** (on the observation covariance matrices). *From the previous properties, it is clear that the observation process $\left\{z_{k};\;k\geq 1\right\}$ is a zero-mean sequence whose covariance function, $\Sigma_{k,s}^{z}\equiv E\left[z_{k}z_{s}^{T}\right]$, is obtained by the following expression:*

(5) $$\Sigma_{k,s}^{z}=E\left[H_{k}A_{k}B_{s}^{T}H_{s}^{T}\right]+R_{k}\delta_{k,s},\;\;\;s\leq k,$$

*where $E\left[H_{k}A_{k}B_{s}^{T}H_{s}^{T}\right]$ and $R_{k}$ are calculated as it is indicated in properties (P1) and (P2), respectively.*

### 3.2. Innovation Approach to the LS Linear Estimation Problem

The proposed covariance-based recursive algorithms for the LS linear prediction, filtering and fixed-point smoothing estimators will be derived by an innovation approach. This approach consists of transforming the observation process $\left\{y_{k};\;k\geq 1\right\}$ into an equivalent one of orthogonal vectors called an innovation process, which will be denoted $\left\{\mu_{k};\;k\geq 1\right\}$ and defined by $\mu_{k}=y_{k}-{\hat{y}}_{k/k-1}$, where ${\hat{y}}_{k/k-1}$ is the orthogonal projection of $y_{k}$ onto the linear space spanned by $\left\{\mu_{1},\ldots ,\mu_{k-1}\right\}$. Since both processes span the same linear subspace, the LS linear estimator of any random vector $\alpha_{k}$ based on the observations $\left\{y_{1},\ldots ,y_{N}\right\}$, denoted by ${\hat{\alpha }}_{k/N}$, is equal to that based on the innovations $\left\{\mu_{1},\ldots ,\mu_{N}\right\}$, and, denoting $\Pi_{h}=E\left[\mu_{h}\mu_{h}^{T}\right]$, the following general expression for the LS linear estimators of $\alpha_{k}$ is obtained

(6) $${\hat{\alpha }}_{k/N}=\sum_{h=1}^{N}E\left[\alpha_{k}\mu_{h}^{T}\right]\Pi_{h}^{-1}\mu_{h}.$$

Hence, to obtain the signal estimators, it is necessary to find an explicit formula beforehand for the innovations and their covariance matrices.

*Innovation* $\mu_{L}$ *and Covariance Matrix* $\Pi_{L}$. Applying orthogonal projections in Equation (3), it is clear that the innovation $\mu_{L}$ is given by

(7) $$\mu_{L}=y_{L}-\;\;\;\sum_{d=0}^{(L-1)\wedge D}\;{\bar{\Gamma }}_{d,L}{\hat{z}}_{L-d/L-1},\;\;L\geq 2;\;\;\;\;\mu_{1}=y_{1},$$

so it is necessary to obtain the one-stage predictor ${\hat{z}}_{L/L-1}$ and the estimators ${\hat{z}}_{L-d/L-1}$, for d=1,…,(L−1)∧D, of the observation process.

In order to obtain the covariance matrix $\Pi_{L}$, we use Equation (3) to express the innovations as:

(8) $$\mu_{L}=\sum_{d=0}^{(L-1)\wedge D}\left(\left(\Gamma_{d,L}-{\bar{\Gamma }}_{d,L}\right)z_{L-d}+{\bar{\Gamma }}_{d,L}\left(z_{L-d}-{\hat{z}}_{L-d/L-1}\right)\right)+w_{L},\;\;L\geq 2$$

and, taking into account that

$$E\left[\left(\Gamma_{d,L}-{\bar{\Gamma }}_{d,L}\right)z_{L-d}{\left(z_{L-d^{\prime }}-{\hat{z}}_{L-d^{\prime }/L-1}\right)}^{T}{\bar{\Gamma }}_{d^{\prime },L}\right]=0,\;\;\forall d,d^{\prime },$$

we have

(9) $$\begin{array}{c} \Pi_{L}=\sum_{d,d^{\prime }=0}^{(L-1)\wedge D}\left(\Sigma_{d,d^{\prime }}^{\gamma_{L}}\circ \Sigma_{L-d,L-d^{\prime }}^{z}+{\bar{\Gamma }}_{d,L}P_{L-d,L-d^{\prime }/L-1}^{z}{\bar{\Gamma }}_{d^{\prime },L}\right)+Q_{L},\;\;L\geq 2;\\ \Pi_{1}=\left(\Sigma_{0,0}^{\gamma_{1}}+{\bar{\gamma }}_{0,1}{\bar{\gamma }}_{0,1}^{T}\right)\circ \Sigma_{1,1}^{z}+Q_{1}, \end{array}$$

where the matrices $\Sigma_{d,d^{\prime }}^{\gamma_{L}}$ and $\Sigma_{L-d,L-d^{\prime }}^{z}$ are given in the Equations (4) and (5), respectively, and $P_{L-d,L-d^{\prime }/L-1}^{z}\equiv E\left[\left(z_{L-d}-{\hat{z}}_{L-d/L-1}\right){\left(z_{L-d^{\prime }}-{\hat{z}}_{L-d^{\prime }/L-1}\right)}^{T}\right]$.

## 4. Least-Squares Linear Signal Estimators

In this section, we derive recursive algorithms for the LS linear estimators, ${\hat{x}}_{k/L},\;k\geq 1,$ of the signal $x_{k}$ based on the observations $\left\{y_{1},\ldots ,y_{L}\right\}$ given in Equation (3); namely, a prediction and filtering algorithm (L≤k) and a fixed-point smoothing algorithm (*k* fixed and L>k) are designed.

### 4.1. Signal Predictor and Filter ${\hat{x}}_{k/L},\;L\leq k$

From the general expression given in Equation (6), to obtain the LS linear estimators ${\hat{x}}_{k/L}=\sum_{h=1}^{L}E\left[x_{k}\mu_{h}^{T}\right]\Pi_{h}^{-1}\mu_{h}$, L≤k, it is necessary to calculate the coefficients

$$X_{k,h}\equiv E\left[x_{k}\mu_{h}^{T}\right]=E\left[x_{k}y_{h}^{T}\right]-E\left[x_{k}{\hat{y}}_{h/h-1}^{T}\right],\;\;h\leq k.$$

- On the one hand, using Equation (3) together with the independence hypotheses and assumption (A1) on the signal covariance factorization, it is clear that
  $$E\left[x_{k}y_{h}^{T}\right]=\;\sum_{d=0}^{(h-1)\wedge D}\;E\left[x_{k}{\left(H_{h-d}x_{h-d}+v_{h-d}\right)}^{T}\right]{\bar{\Gamma }}_{d,h}=A_{k}\;\sum_{d=0}^{(h-1)\wedge D}\;B_{h-d}^{T}{\bar{H}}_{h-d}^{T}{\bar{\Gamma }}_{d,h},\;\;\;h\leq k.$$
- On the other hand, since ${\hat{y}}_{h/h-1}=\;\sum_{d=0}^{(h-1)\wedge D}{\bar{\Gamma }}_{d,h}{\hat{z}}_{h-d/h-1},\;h\geq 2,$ and taking into account that, from Equation (6), ${\hat{z}}_{h-d/h-1}=\sum_{j=1}^{h-1}Z_{h-d,j}\Pi_{j}^{-1}\mu_{j}$ with $Z_{h-d,j}=E\left[z_{h-d}\mu_{j}^{T}\right]$, the following identity holds
  $$E\left[x_{k}{\hat{y}}_{h/h-1}^{T}\right]=\sum_{d=0}^{(h-1)\wedge D}\;\;\left(\sum_{j=1}^{h-1}X_{k,j}\Pi_{j}^{-1}Z_{h-d,j}^{T}\right){\bar{\Gamma }}_{d,h}.$$

Therefore, it is easy to check that $X_{k,h}=A_{k}E_{h},\;1\leq h\leq k,$ where $E_{h}$ is a function satisfying that

(10) $$\begin{array}{c} E_{h}=\;\;\sum_{d=0}^{(h-1)\wedge D}\;B_{h-d}^{T}{\bar{H}}_{h-d}^{T}{\bar{\Gamma }}_{d,h}-\;\;\sum_{d=0}^{(h-1)\wedge D}\;\;\left(\sum_{j=1}^{h-1}E_{j}\Pi_{j}^{-1}Z_{h-d,j}^{T}\right){\bar{\Gamma }}_{d,h},\;\;h\geq 2,\\ E_{1}=B_{1}^{T}{\bar{H}}_{1}^{T}{\bar{\Gamma }}_{0,1}. \end{array}$$

Hence, it is clear that the signal prediction and filtering estimators can be expressed as

(11) $${\hat{x}}_{k/L}=A_{k}e_{L},\;\;L\leq k,\;\;k\geq 1,$$

where the vectors $e_{L}$ are defined by $e_{L}=\sum_{h=1}^{L}E_{h}\Pi_{h}^{-1}\mu_{h}$, for L≥1, with $e_{0}=0$, thus obeying the recursive relation

(12) $$e_{L}=e_{L-1}+E_{L}\Pi_{L}^{-1}\mu_{L},\;\;L\geq 1;\;\;\;\;e_{0}=0.$$

*Matrices $E_{L}$.* Taking into account the above relation, an expression for $E_{L},\;L\geq 1,$ must be derived. For this purpose, Equation (10) is rewritten for h=L as

$$E_{L}=\sum_{d=0}^{(L-1)\wedge D}\;B_{L-d}^{T}{\bar{H}}_{L-d}^{T}{\bar{\Gamma }}_{d,L}-\;\;\sum_{d=0}^{(L-1)\wedge D}\;\left(\sum_{j=1}^{L-1}E_{j}\Pi_{j}^{-1}Z_{L-d,j}^{T}\right){\bar{\Gamma }}_{d,L},\;\;L\geq 2,$$

and we examine the cases d=0 and d≥1 separately:

− For d=0, using Equation (3), it holds that $Z_{L,j}={\bar{H}}_{L}X_{L,j}={\bar{H}}_{L}A_{L}E_{j}$, for j<L, and, by denoting $\Sigma_{L}^{e}=\sum_{h=1}^{L}E_{h}\Pi_{h}^{-1}E_{h}$, L≥1, we obtain that
  $$\left(\sum_{j=1}^{L-1}E_{j}\Pi_{j}^{-1}Z_{L,j}^{T}\right){\bar{\Gamma }}_{0,L}=\left(\sum_{j=1}^{L-1}E_{j}\Pi_{j}^{-1}E_{j}^{T}\right)A_{L}^{T}{\bar{H}}_{L}^{T}{\bar{\Gamma }}_{0,L}=\Sigma_{L-1}^{e}A_{L}^{T}{\bar{H}}_{L}^{T}{\bar{\Gamma }}_{0,L}.$$
− For d≥1, since $Z_{L-d,j}={\bar{H}}_{L-d}A_{L-d}E_{j}$, for j<L−d, we can see that
  $$\left(\sum_{j=1}^{L-1}E_{j}\Pi_{j}^{-1}Z_{L-d,j}^{T}\right){\bar{\Gamma }}_{d,L}=\Sigma_{L-d-1}^{e}A_{L-d}^{T}{\bar{H}}_{L-d}^{T}{\bar{\Gamma }}_{d,L}+\left(\sum_{j=L-d}^{L-1}E_{j}\Pi_{j}^{-1}Z_{L-d,j}^{T}\right){\bar{\Gamma }}_{d,L}.$$

By substituting the above sums in $E_{L}$, it is deduced that

(13) $$\;\begin{array}{c} E_{L}=\;\;\;\sum_{d=0}^{(L-1)\wedge D}\;{\left(B_{L-d}-A_{L-d}\Sigma_{L-d-1}^{e}\right)}^{T}{\bar{H}}_{L-d}^{T}{\bar{\Gamma }}_{d,L}-\;\;\;\sum_{d=1}^{(L-1)\wedge D}\;\left(\sum_{j=L-d}^{L-1}E_{j}\Pi_{j}^{-1}Z_{L-d,j}^{T}\right){\bar{\Gamma }}_{d,L},\;\;L\geq 2;\\ E_{1}=B_{1}^{T}{\bar{H}}_{1}^{T}{\bar{\Gamma }}_{0,1}, \end{array}$$

where the matrices $Z_{L-d,j},\;j\geq L-d$, will be obtained in the next subsection, as they correspond to the observation smoothing estimators, and the matrices $\Sigma_{L}^{e}$ are recursively obtained by

(14) $$\Sigma_{L}^{e}=\Sigma_{L-1}^{e}+E_{L}\Pi_{L}^{-1}E_{L}^{T},\;\;L\geq 1;\;\;\Sigma_{0}^{e}=0.$$

Finally, from assumption (A1) and since the estimation errors are orthogonal to the estimators, we have that the error covariance matrices, $P_{k/L}^{x}\equiv E\left[\left(x_{k}-{\hat{x}}_{k/L}\right){\left(x_{k}-{\hat{x}}_{k/L}\right)}^{T}\right]$, are given by

(15) $$P_{k/L}^{x}=A_{k}{\left(B_{k}-A_{k}\Sigma_{L}^{e}\right)}^{T}\;,\;\;L\leq k,\;\;k\geq 1.$$

### 4.2. Estimators of the Observations ${\hat{z}}_{k/L},\;k\geq 1$

As it has been already indicated, the Equation (7) require obtaining the observation estimators (predictor, filter and smoother). From the general expression for the estimators given in Equation (6), we have that ${\hat{z}}_{k/L}=\sum_{j=1}^{L}Z_{k,j}\Pi_{j}^{-1}\mu_{j}$, with $Z_{k,j}=E\left[z_{k}\mu_{j}^{T}\right]$. Next, recursive expressions will be derived separately for L<k (predictors) and L≥k (filter and smoothers).

*Observation Prediction Estimators.* Since $Z_{k,j}={\bar{H}}_{k}A_{k}E_{j}$, for j<k, we have that the prediction estimators of the observations are given by

(16) $${\hat{z}}_{k/L}={\bar{H}}_{k}A_{L}e_{L},\;\;L<k,\;\;k\geq 1.$$

*Observation Filtering and Fixed-Point Smoothing Estimators.* Clearly, the filter and fixed-point smoothers of the observations are obtained by the following recursive expression:

(17) $${\hat{z}}_{k/L}={\hat{z}}_{k/L-1}+Z_{k,L}\Pi_{L}^{-1}\mu_{L},\;\;L\geq k,\;\;k\geq 1,$$

with initial condition given by the one-stage predictor ${\hat{z}}_{k/k-1}={\bar{H}}_{k}A_{k}e_{k-1}$.

Hence, the matrices $Z_{k,L}$ must be calculated for L≥k. Since the innovation is a white process, $E\left[{\hat{z}}_{k/L-1}\mu_{L}^{T}\right]=0$ and hence $Z_{k,L}=E\left[z_{k}\mu_{L}^{T}\right]=E\left[\left(z_{k}-{\hat{z}}_{k/L-1}\right)\mu_{L}^{T}\right]$. Now using Equation (8) for $\mu_{L}$ and, taking into account that $E\left[\left(z_{k}-{\hat{z}}_{k/L-1}\right)z_{L-d}^{T}\left(\Gamma_{d,L}-{\bar{\Gamma }}_{d,L}\right)\right]=0,\;\;\forall d,$ we have

(18) $$Z_{k,L}=\sum_{d=0}^{(L-1)\wedge D}P_{k,L-d/L-1}^{z}{\bar{\Gamma }}_{d,L},\;\;L\geq k,$$

where $P_{k,L-d/L-1}^{z}\equiv E\left[\left(z_{k}-{\hat{z}}_{k/L-1}\right){\left(z_{L-d}-{\hat{z}}_{L-d/L-1}\right)}^{T}\right]$.

Consequently, the error covariance matrices $P_{k,h/m}^{z}=E\left[\left(z_{k}-{\hat{z}}_{k/m}\right){\left(z_{h}-{\hat{z}}_{h/m}\right)}^{T}\right]$ must be derived, for which the following two cases are analyzed separately:

* For m≥k∧h, using Equation (17) and taking into account that $Z_{k,m}=E\left[\left(z_{k}-{\hat{z}}_{k/m-1}\right)\mu_{m}^{T}\right]$, it is easy to see that
  (19) $$P_{k,h/m}^{z}=P_{k,h/m-1}^{z}-Z_{k,m}\Pi_{m}^{-1}Z_{h,m}^{T},\;\;\;m\geq k\wedge h.$$
* For m<h≤k, using Equation (16), assumption (A1) and the orthogonality between the estimation errors and the estimators, we obtain
  (20) $$P_{k,h/m}^{z}=A_{k}B_{h}^{T}-{\bar{H}}_{k}A_{k}\Sigma_{m}^{e}A_{h}^{T}{\bar{H}}_{h}^{T},\;\;\;m<h\leq k.$$

### 4.3. Signal Fixed-Point Smoother ${\hat{x}}_{k/L},\;L>k$

Starting with the filter, ${\hat{x}}_{k/k}$, and the filtering error covariance matrix, $P_{k/k}^{x}$, it is clear that the signal fixed-point smoother ${\hat{x}}_{k/L}$, L>k, and the corresponding error covariance matrix, $P_{k/L}^{x}\equiv E\left[\left(x_{k}-{\hat{x}}_{k/L}\right){\left(x_{k}-{\hat{x}}_{k/L}\right)}^{T}\right]$, are obtained by

(21) $$\begin{array}{c} {\hat{x}}_{k/L}={\hat{x}}_{k/L-1}+X_{k,L}\Pi_{L}^{-1}\mu_{L},\;\;L>k,\;\;k\geq 1,\\ P_{k/L}^{x}=P_{k/L-1}^{x}-X_{k,L}\Pi_{L}^{-1}X_{k,L}^{T},\;\;L>k,\;\;k\geq 1. \end{array}$$

An analogous reasoning to that of Equation (18) leads to the following expression for the matrices $X_{k,L}$:

(22) $$X_{k,L}=\sum_{d=0}^{(L-1)\wedge D}P_{k,L-d/L-1}^{xz}{\bar{\Gamma }}_{d,L},\;\;L>k,$$

where $P_{k,L-d/L-1}^{xz}\equiv E\left[\left(x_{k}-{\hat{x}}_{k/L-1}\right){\left(z_{L-d}-{\hat{z}}_{L-d/L-1}\right)}^{T}\right]$.

The derivation of the error cross-covariance matrices $P_{k,h/m}^{xz}=E\left[\left(x_{k}-{\hat{x}}_{k/m}\right){\left(z_{h}-{\hat{z}}_{h/m}\right)}^{T}\right]$ is similar to that of the matrices $P_{k,h/m}^{z}$, and they are given by

(23) $$\begin{array}{cc} P_{k,h/m}^{xz}=P_{k,h/m-1}^{xz}-X_{k,m}\Pi_{m}^{-1}Z_{h,m}^{T}, & \;\;m\geq k\wedge h,\\ P_{k,h/m}^{xz}=A_{k}{\left(B_{h}-A_{h}\Sigma_{m}^{e}\right)}^{T}H_{h}^{T}, & \;\;m<h\leq k,\\ P_{k,h/m}^{xz}=\left(B_{k}-A_{k}\Sigma_{m}^{e}\right)A_{h}^{T}H_{h}^{T}, & \;\;m<k\leq h, \end{array}$$

where $X_{k,m}=A_{k}E_{m}$, for m≤k, and $Z_{h,m}={\bar{H}}_{h}A_{m}E_{m}$, for m<h, and otherwise these matrices are given by Equations (22) and (18), respectively.

### 4.4. Recursive Algorithms: Computational Procedure

The computational procedure of the proposed prediction, filtering and fixed-point smoothing algorithms can be summarized as follows:

(1) *Covariance Matrices.* The covariance matrices $\Sigma_{d,d^{\prime }}^{\gamma_{k}}$ and $\Sigma_{k}^{z}$ are obtained by Equations (4) and (5), respectively; these matrices only depend on the system model information, so they can be calculated offline before the observed data packets are available.
(2) *LS Linear Prediction and Filtering Recursive Algorithm.* At the sampling time *k*, once the (k−1)-th iteration is finished and $E_{k-1}$, $\Pi_{k-1}$, $\Sigma_{k-1}^{e}$$\mu_{k-1}$ and $e_{k-1}$ are known, the proposed prediction and filtering algorithm operates as follows:
  (2a) Compute $Z_{k,k-1}={\bar{H}}_{k}A_{k}E_{k-1}$ and $Z_{k-d,k-1}$, for d=1,…,(k−1)∧D, by Equation (18). From these matrices, we obtain the observation estimators ${\hat{z}}_{k-d/k-1}$, for d=0,1,…,(k−1)∧D, by Equation (19) and (20), and the observation error covariance matrices $P_{k-d,k-d^{\prime }/k-1}^{z}$, for $d,d^{\prime }=0,1,\ldots ,\left(k-1\right)\wedge D$, by Equation (19) and (20).
  (2b) Compute $E_{k}$ by Equation (13) and use $P_{k-d,k-d^{\prime }/k-1}^{z}$ to obtain the innovation covariance matrix $\Pi_{k}$ by Equation (9). Then, $\Sigma_{k}^{e}$ is obtained by Equation (14) and, from it, the prediction and filtering error covariance matrices, $P_{k/k-s}^{x}$ and $P_{k/k}^{x}$, respectively, are obtained by Equation (15).
  (2c) When the new measurement $y_{k}$ is available, the innovation $\mu_{k}$ is computed by Equation (7) using ${\hat{z}}_{k-d/k-1}$, for d=0,1,…,(k−1)∧D, and, from the innovation, $e_{k}$ is obtained by Equation (12). Then, the predictors, ${\hat{x}}_{k/k-s}$ and the filter, ${\hat{x}}_{k/k}$ are computed by Equation (11).
(3) *LS linear fixed-point smoothing recursive algorithm.* Once the filter, ${\hat{x}}_{k/k}$, and the filtering error covariance matrix, $P_{k/k}^{x}$ are available, the proposed smoothing estimators and the corresponding error covariance matrix are obtained as follows:
  For L=k+1,k+2,…, compute the error cross-covariance matrices $P_{k,L-d/L-1}^{xz}$, for d=0,1,…,(k−1)∧D, using Equation (23) and, from these matrices, $X_{k,L}$ is derived by Equation (22); then, the smoothers ${\hat{x}}_{k/L}$ and their error covariance matrices $P_{k/L}^{x}$ are obtained from Equation (21).

## 5. Computer Simulation Results

In this section, a numerical example is presented with the following purposes: (a) to show that, although the current covariance-based estimation algorithms do not require the evolution model generating the signal process, they are also applicable to the conventional formulation using the state-space model, even in the presence of state-dependent multiplicative noise; (b) to illustrate some kinds of uncertainties which can be covered by the current model with random measurement matrices; and (c) to analyze how the estimation accuracy of the proposed algorithms is influenced by the sensor uncertainties and the random delays in the transmissions.

*Signal Evolution Model with State-Dependent Multiplicative Noise.* Consider a scalar signal $\left\{x_{k};\;k\geq 0\right\}$ whose evolution is given by the following model with multiplicative and additive noises:

$$x_{k}=\left(0.9+0.01\epsilon_{k-1}\right)x_{k-1}+\xi_{k-1},\;k\geq 1,$$

where $x_{0}$ is a standard Gaussian variable and $\left\{\epsilon_{k};\;k\geq 0\right\}$, $\left\{\xi_{k};\;k\geq 0\right\}$ are zero-mean Gaussian white processes with unit variance. Assuming that $x_{0}$, $\left\{\epsilon_{k};\;k\geq 0\right\}$ and $\left\{\xi_{k};\;k\geq 0\right\}$ are mutually independent, the signal covariance function is given by $E\left[x_{k}x_{s}\right]=0.9^{k-s}D_{s},\;s\leq k,$ where $D_{s}=E\left[x_{s}^{2}\right]$ is recursively obtained by $D_{s}=0.8101D_{s-1}+1,$ for s≥1, with $D_{0}=1;$ hence, the signal process satisfies assumption *(A1)* taking, for example, $A_{k}=0.9^{k}$ and $B_{s}=0.9^{-s}D_{s}$.

*Sensor Measured Outputs.* As in [22], let us consider scalar measurements provided by four sensors with different types of uncertainty: continuous and discrete gain degradation in sensors 1 and 2, respectively, missing measurements in sensor 3, and both missing measurements and multiplicative noise in sensor 4. These uncertainties can be described in a unified way by the current model with random measurement matrices; specifically, the measured outputs are described according to Equation (1):

$$z_{k}^{\left(i\right)}=H_{k}^{\left(i\right)}x_{k}+v_{k}^{\left(i\right)},\;k\geq 1,\;i=1,2,3,4,$$

with the following characteristics:

- For i=1,2,3, $H_{k}^{\left(i\right)}=C^{\left(i\right)}\theta_{k}^{\left(i\right)}$ and $H_{k}^{\left(4\right)}=\left(C^{\left(4\right)}+C^{\left(4^{\prime }\right)}\rho_{k}\right)\theta_{k}^{\left(4\right)}$, where $C^{\left(1\right)}=C^{\left(3\right)}=0.8$, $C^{\left(2\right)}=C^{\left(4\right)}=0.75$, $C^{\left(4^{\prime }\right)}=0.95$, and $\left\{\rho_{k};\;k\geq 1\right\}$ is a zero-mean Gaussian white process with unit variance. The sequences $\left\{\rho_{k};\;k\geq 1\right\}$ and $\{\theta_{k}^{\left(i\right)};\;k\geq 1\},\;i=1,2,3,4,$ are mutually independent, and $\{\theta_{k}^{\left(i\right)};\;k\geq 1\},\;i=1,2,3,4$, are white processes with the following time-invariant probability distributions:
  − $\theta_{k}^{\left(1\right)}$ is uniformly distributed over the interval [0.1,0.9];
  − $P\left[\theta_{k}^{\left(2\right)}=0\right]=0.3,\;\;P\left[\theta_{k}^{\left(2\right)}=0.5\right]=0.3\;\;P\left[\theta_{k}^{\left(2\right)}=1\right]=0.4;$
  − For i=3,4, $\theta_{k}^{\left(i\right)}$ are Bernoulli random variables with the same time-invariant probabilities in both sensors $P[\theta_{k}^{\left(i\right)}=1]=p$.
- The additive noises are defined by $v_{k}^{\left(i\right)}=c_{i}\eta_{k}^{v},\;i=1,2,3,4$, where $c_{1}=0.5$, $c_{2}=c_{3}=0.75$, $c_{4}=1$, and $\left\{\eta_{k}^{v};\;k\geq 1\right\}$ is a zero-mean Gaussian white process with unit variance. Clearly, the additive noises $\left\{v_{k}^{\left(i\right)};\;k\geq 1\right\}$, i=1,2,3,4, are correlated at any time, with $R_{k}^{\left(ij\right)}=c_{i}c_{j},\;i,j=1,2,3,4.$

*Observations with Bounded Random Delays and Packet Dropouts.* Next, according to the theoretical observation model, let us suppose that bounded random measurement delays and packet dropouts, with different delay probabilities, exist in the data transmission. Specifically, assuming that the largest delay is D=3, let us consider the observation Equation (2):

$$y_{k}^{\left(i\right)}=\sum_{d=0}^{(k-1)\wedge 3}\gamma_{d,k}^{\left(i\right)}z_{k-d}^{\left(i\right)}+w_{k}^{\left(i\right)},\;\;k\geq 1,$$

where, for i=1,2,3,4 and d=0,1,2,3, $\left\{\gamma_{d,k}^{\left(i\right)};\;k>d\right\}$, are sequences of independent Bernoulli variables with the same time-invariant delay probabilities for the four sensors ${\bar{\gamma }}_{d,k}^{\left(i\right)}={\bar{\gamma }}_{d}$, where $\sum_{d=0}^{3}{\bar{\gamma }}_{d}\leq 1$. Hence, the packet dropout probability is $1-\sum_{d=0}^{3}{\bar{\gamma }}_{d}$. The transmission noise is defined by $w_{k}^{\left(i\right)}=c_{i}\eta_{k}^{w}$, i=1,2,3,4, where $\left\{\eta_{k}^{w};\;k\geq 1\right\}$ is a zero-mean Gaussian white process with unit variance.

Finally, in order to apply the proposed algorithms, and according to (A5), we will assume that all of the processes involved in the observation equations are mutually independent.

To illustrate the feasibility and effectiveness of the proposed algorithms, they were implemented in MATLAB (R2011b 7.13.0.564, The Mathworks, Natick, MA, USA) and one hundred iterations of the prediction, filtering and fixed-point smoothing algorithms have been performed. In order to analyze the effect of the network-induced uncertainties on the estimation accuracy, different values of the probabilities *p* of the Bernoulli random variables that model the uncertainties of the third and fourth sensors, and several values of the delay probabilities ${\bar{\gamma }}_{d}$, d=0,1,2,3, have been considered.

**Figure 1:**
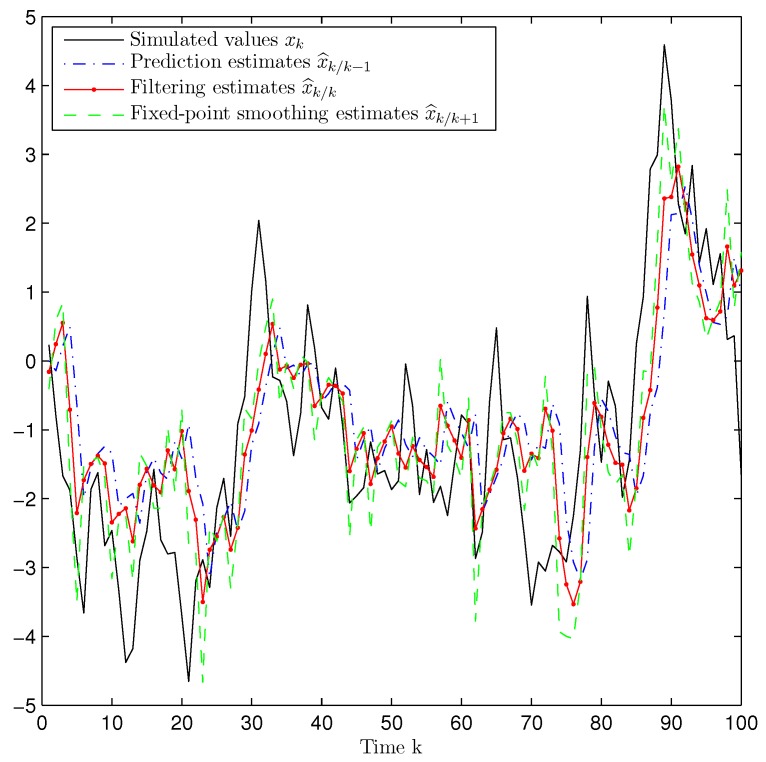
Simulated signal and proposed prediction, filtering and smoothing estimates when p=0.5 and ${\bar{\gamma }}_{0}=0.6$, ${\bar{\gamma }}_{d}=0.1,\;d=1,2,3$.

**Figure 2:**
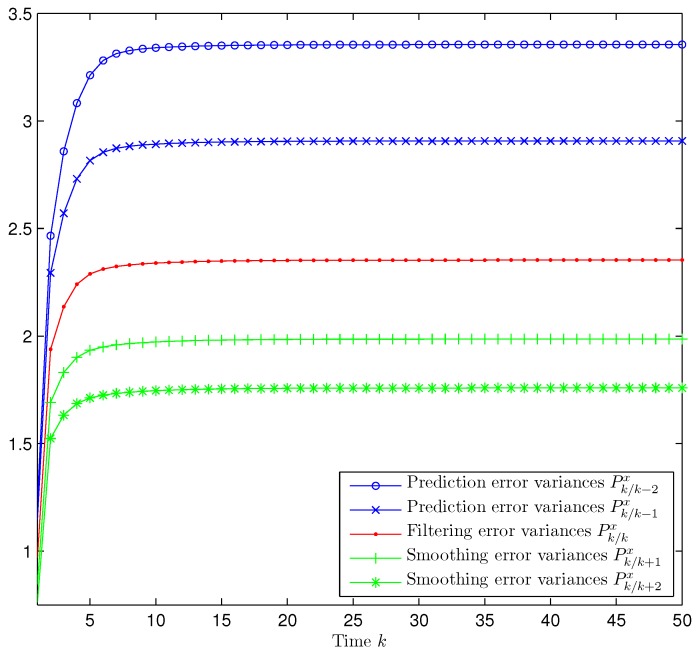
Prediction, filtering and smoothing error variances when p=0.5 and ${\bar{\gamma }}_{0}=0.6$, ${\bar{\gamma }}_{d}=0.1$, *d* = 1,2,3.

**Figure 3:**
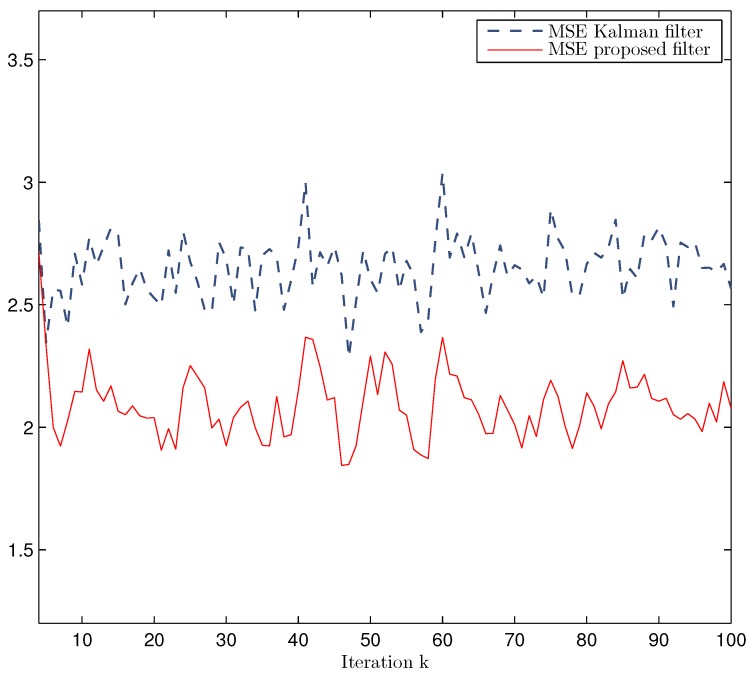
MSE of Kalman filter and proposed filter when p=0.5 and ${\bar{\gamma }}_{0}=0.6$, ${\bar{\gamma }}_{d}=0.1,\;d=1,2,3$.

*Performance of the Prediction, Filtering and Fixed-Point Smoothing Estimators.* Considering the values p=0.5, ${\bar{\gamma }}_{0}=0.6$ (packet arrival probability) and ${\bar{\gamma }}_{d}=\frac{1-{\bar{\gamma }}_{0}}{4},\;d=1,2,3$ (delay probabilities), Figure 1 displays a simulated trajectory along with the prediction, filtering and smoothing estimations, showing a satisfactory and efficient tracking performance of the proposed estimators. Figure 2 shows the error variances of the predictors ${\hat{z}}_{k/k-2}$ and ${\hat{z}}_{k/k-1}$, the filter ${\hat{z}}_{k/k}$ and the smoothers ${\hat{z}}_{k/k+1}$ and ${\hat{z}}_{k/k+2}$. Analogously to what happens for non-delayed observations, the performance of the estimators becomes better as more observations are used; that is, the error variances of the smoothing estimators are less than the filtering ones which, in turn, are less than those of the predictors. Hence, the estimation accuracy of the smoothers is superior to that of the filter and predictors and improves as the number of iterations in the fixed-point smoothing algorithm increases. The performance of the proposed filter has also been evaluated in comparison with the standard Kalman filter; for this purpose, the filtering mean square error (MSE) at each sampling time was calculated by considering one thousand independent simulations and one hundred iterations of each filter. The results of this comparison are displayed in Figure 3, which shows that the proposed filter performs better than the Kalman filter, a fact that was expected since the latter does not take into account either the uncertainties in the measured outputs or the delays and losses during transmission.

**Figure 4:**
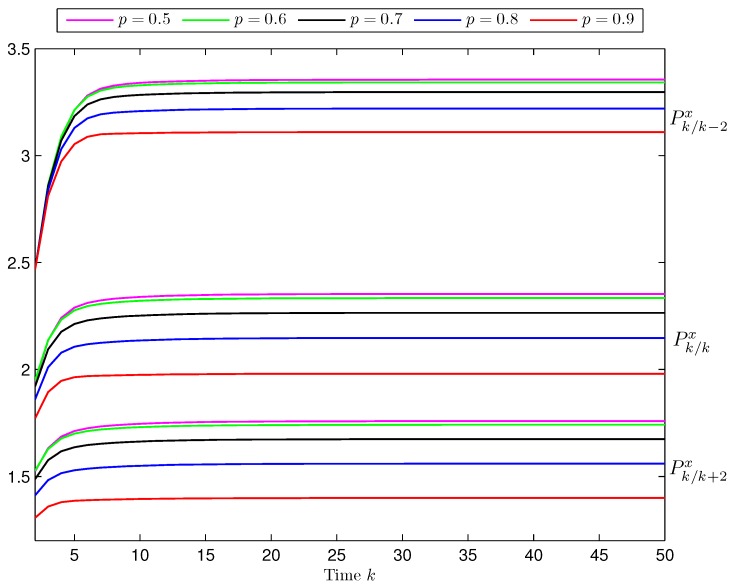
Prediction, filtering and fixed-point smoothing error variances for different values of the probability *p*, when ${\bar{\gamma }}_{0}=0.6$ and ${\bar{\gamma }}_{d}=0.1,\;d=1,2,3$.

*Influence of the Missing Measurements.* To analyze the sensitivity of the estimation performance on the effect of the missing measurements phenomenon in the third and fourth sensors, the error variances are calculated for different values of the probability *p*. Specifically, considering again ${\bar{\gamma }}_{0}=0.6$ and ${\bar{\gamma }}_{d}=0.1,\;d=1,2,3,$ Figure 4 displays the prediction, filtering and fixed-point smoothing error variances for the values *p* = 0.5 to *p* = 0.9. This Figure shows that, as *p* increases, the estimation error variances become smaller and, hence, as it was expected, the performance of the estimators improves as the probability of missing measurements 1−p decreases.

**Figure 5:**
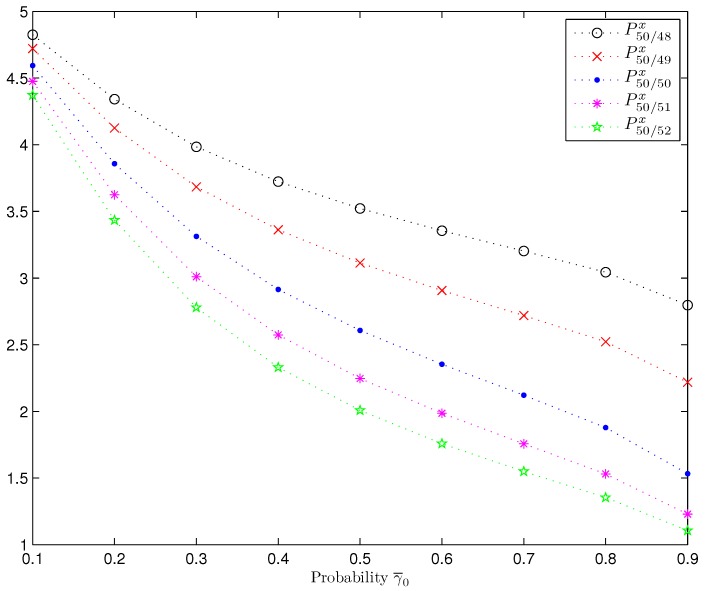
Estimation error variances for different values of the probability ${\bar{\gamma }}_{0}$, when p=0.5.

*Influence of the Transmission Random Delays and Packet Dropouts.* Considering a fixed value of the probability *p*, namely, p=0.5, in order to show how the estimation accuracy is influenced by the transmission random delays and packet dropouts, the prediction, filtering and smoothing error variances are displayed in Figure 5 when ${\bar{\gamma }}_{0}$ is varied from 0.1 to 0.9, considering again ${\bar{\gamma }}_{d}=\frac{1-{\bar{\gamma }}_{0}}{4},\;d=1,2,3$. Since the behaviour of the error variances is analogous from a certain iteration on, only the results at the iteration k=50 are shown in Figure 5. From this figure, we see that the performance of the estimators (predictor, filter and smoother) is indeed influenced by the transmission delay and packet dropout probabilities and, as it was expected, it is confirmed that the error variances become smaller, and hence the performance of the estimators improves, as the packet arrival probability ${\bar{\gamma }}_{0}$ increases. Moreover, for the filter and the smoothers, this improvement is more significant than for the predictors. In addition, as it was deduced from Figure 2, it is observed that, for all the values of ${\bar{\gamma }}_{0}$, the performance of the estimators is better as more observations are used, and this improvement is more significant as ${\bar{\gamma }}_{0}$ increases.

Finally, it must be remarked that analogous conclusions are deduced for other values of the probabilities *p* and ${\bar{\gamma }}_{d},\;d=0,1,2,3$, and also when such probabilities are different at the different sensors.

## 6. Conclusions

This paper makes valuable contributions to the optimal fusion estimation problem in networked stochastic systems with random parameter matrices, when multi-step delays or even packet dropouts occur randomly during the data transmission. By an innovation approach, recursive prediction, filtering and fixed-point smoothing algorithms have been designed, which are easily implementable and do not require the signal evolution model, but only the mean and covariance functions of the system processes.

Unlike other estimation algorithms proposed in the literature, where the estimators are restricted to obey a particular structure, in this paper, recursive optimal estimation algorithms are designed without requiring a particular structure on the estimators, but just using the LS optimality criterion. Another advantage is that the current approach does not resort to the augmentation technique and, consequently, the dimension of the designed estimators is the same as that of the original signal, thus reducing the computational burden in the processing centre.

## References

[1] Ran C., Deng Z. (2012). Self-tuning weighted measurement fusion Kalman filtering algorithm. Comput. Stat. Data Anal..

[2] Feng J., Zeng M. (2012). Optimal distributed Kalman filtering fusion for a linear dynamic system with cross-correlated noises. Int. J. Syst. Sci..

[3] Yan L., Li X., Xia Y., Fu M. (2013). Optimal sequential and distributed fusion for state estimation in cross-correlated noise. Automatica.

[4] Ma J., Sun S. (2013). Centralized fusion estimators for multisensor systems with random sensor delays, multiple packet dropouts and uncertain observations. IEEE Sens. J..

[5] Gao S., Chen P. (2014). Suboptimal filtering of networked discrete-time systems with random observation losses. Math. Probl. Eng..

[6] Liu Y., He X., Wang Z., Zhou D. (2014). Optimal filtering for networked systems with stochastic sensor gain degradation. Automatica.

[7] Chen B., Zhang W., Yu L. (2014). Distributed fusion estimation with missing measurements, random transmission delays and packet dropouts. IEEE Trans. Autom. Control.

[8] Li N., Sun S., Ma J. (2014). Multi-sensor distributed fusion filtering for networked systems with different delay and loss rates. Digit. Signal Process..

[9] Wang S., Fang H., Tian X. (2015). Recursive estimation for nonlinear stochastic systems with multi-step transmission delays, multiple packet dropouts and correlated noises. Signal Process..

[10] Chen D., Yu Y., Xu L., Liu X. (2015). Kalman filtering for discrete stochastic systems with multiplicative noises and random two-step sensor delays. Discret. Dyn. Nat. Soc..

[11] García-Ligero M.J., Hermoso-Carazo A., Linares-Pérez J. (2015). Distributed fusion estimation in networked systems with uncertain observations and markovian random delays. Signal Process..

[12] Gao S., Chen P., Huang D., Niu Q. (2016). Stability analysis of multi-sensor Kalman filtering over lossy networks. Sensors.

[13] Lin H., Sun S. (2016). State estimation for a class of non-uniform sampling systems with missing measurements. Sensors.

[14] Hu J., Wang Z., Chen D., Alsaadi F.E. (2016). Estimation, filtering and fusion for networked systems with network-induced phenomena: New progress and prospects. Inf. Fusion.

[15] Sun S., Lin H., Ma J., Li X. (2017). Multi-sensor distributed fusion estimation with applications in networked systems: A review paper. Inf. Fusion.

[16] Li W., Jia Y., Du J. (2017). Distributed filtering for discrete-time linear systems with fading measurements and time-correlated noise. Digit. Signal Process..

[17] Luo Y., Zhu Y., Luo D., Zhou J., Song E., Wang D. (2008). Globally optimal multisensor distributed random parameter matrices Kalman filtering fusion with applications. Sensors.

[18] Shen X.J., Luo Y.T., Zhu Y.M., Song E.B. (2012). Globally optimal distributed Kalman filtering fusion. Sci. China Inf. Sci..

[19] Hu J., Wang Z., Gao H. (2013). Recursive filtering with random parameter matrices, multiple fading measurements and correlated noises. Automatica.

[20] Linares-Pérez J., Caballero-Águila R., García-Garrido I. (2014). Optimal linear filter design for systems with correlation in the measurement matrices and noises: Recursive algorithm and applications. Int. J. Syst. Sci..

[21] Yang Y., Liang Y., Pan Q., Qin Y., Yang F. (2016). Distributed fusion estimation with square-root array implementation for Markovian jump linear systems with random parameter matrices and cross-correlated noises. Inf. Sci..

[22] Caballero-Águila R., Hermoso-Carazo A., Linares-Pérez J. (2016). Networked fusion filtering from outputs with stochastic uncertainties and correlated random transmission delays. Sensors.

[23] Caballero-Águila R., Hermoso-Carazo A., Linares-Pérez J. (2016). Fusion estimation using measured outputs with random parameter matrices subject to random delays and packet dropouts. Signal Process..

[24] Feng J., Zeng M. (2011). Descriptor recursive estimation for multiple sensors with different delay rates. Int. J. Control.

[25] Caballero-Águila R., Hermoso-Carazo A., Linares-Pérez J. (2012). Least-squares linear-estimators using measurements transmitted by different sensors with packet dropouts. Digit. Signal Process..

[26] Ma J., Sun S. (2017). Distributed fusion filter for networked stochastic uncertain systems with transmission delays and packet dropouts. Signal Process..

[27] Wang S., Fang H., Liu X. (2015). Distributed state estimation for stochastic non-linear systems with random delays and packet dropouts. IET Control Theory Appl..

[28] Sun S. (2009). Linear minimum variance estimators for systems with bounded random measurement delays and packet dropouts. Signal Process..

[29] Sun S. (2013). Optimal linear filters for discrete-time systems with randomly delayed and lost measurements with/without time stamps. IEEE Trans. Autom. Control.

[30] Sun S., Ma J. (2014). Linear estimation for networked control systems with random transmission delays and packet dropouts. Inf. Sci..

[31] Wang S., Fang H., Tian X. (2016). Minimum variance estimation for linear uncertain systems with one-step correlated noises and incomplete measurements. Digit. Signal Process..

[32] Tian T., Sun S., Li N. (2016). Multi-sensor information fusion estimators for stochastic uncertain systems with correlated noises. Inf. Fusion.

